# Some epidemiological aspects of nosocomial infections. Antibiotic sensitivity rates of isolated bacteria from nosocomial infections - A prospective study from 2012 to 2013 in the Academic Emergency Hospital Sibiu, Romania

**DOI:** 10.1186/1471-2334-14-S7-O29

**Published:** 2014-10-15

**Authors:** Victoria Bîrluțiu, Rareş-Mircea Bîrluțiu

**Affiliations:** 1 “Lucian Blaga” University Sibiu, Faculty of Medicine Sibiu, Romania; 2 Academic Emergency Hospital Sibiu, Romania

## Background

The aim of this study was to monitor the nosocomial infections in our hospital (The Academic Emergency Hospital in Sibiu), to monitor the antibiotic sensitivity patterns of isolated bacteria from nosocomial infections and to follow the variation in resistance from 2012 to 2013, in order to establish the first-line therapy for critical patients with nosocomial infections.

## Methods

We collected the data related to hospital service units where nosocomial infections had been identified, the etiologic agents that were identified, the site of infection and the antibiotic sensitivity rates of nosocomial infections.

## Results

The total number of isolated strains was 413, 231 in 2012 and 182 in 2013. In the intensive care units 151 nosocomial infections were identified; 88 strains in the Surgical Department, 27 strains in the Department of Gynecology and Obstetrics, 24 strains in the Department of Neurosurgery and 23 in the Orthopedic Department. 19 strains were identified in the Neurology Department and also in the Internal Medicine Department, 17 strains were identified in the Urology Department, 14 in the Aesthetic Surgery Department, 8 in the Nephrology Department, 8 in the Hematology Department and 5 in the Gastroenterology Department. 3 strains were isolated in each of the following departments: Diabetes and Nutrition Diseases, E.N.T and Neonatology and 1 strain was identified in the Cardiology Department. In terms of etiology, the most commonly isolated were: *Enterobacter* spp. (111) followed by *Acinetobacter* spp. (71), *Escherichia coli* (59), *Staphylococcus aureus* (46), *Klebsiella* spp. (30), *Enterococcus* spp. (20), *Pseudomonas* spp. (19), *Proteus* spp. (19), fungi (17), coagulase-negative *Staphylococcus* (11), *Burkholderia cepacia* (8), *Serratia marcescens* (5).

## Conclusion

Most cases of nosocomial infections were reported from ICUs and surgery, representing surgical wound infections and nosocomial pneumonia. In aspiration pneumonia patients, *Acinetobacter* spp. (22) was isolated the most frequently. In blood cultures the most frequently isolated strains were *Klebsiella pneumoniae* and *Staphylococcus aureus*. *Burkholderia cepacia* was associated with hematologic malignancies. *Enterobacter* spp. was the most common etiologic agent isolated from surgical wound infections and from nosocomial urinary tract infections. The use of broad-spectrum antibiotics was associated with the emergence of carbapenemase in over 90% of the isolated strains of *Acinetobacter* spp. and *Enterobacter* AmpC (+), in 54.05% of *Enterobacter* extended-spectrum beta-lactamase (ESBL +), in 20.34% of the *Escherichia coli* ESBL+ isolated strains. Methicillin-resistant *Staphylococcus aureus* (MRSA) was isolated in 67.74% of all *Staphylococcus aureus* strains.

